# Performance of Urine Fluorescence In Situ Hybridization for Diagnosis of Upper Tract Urothelial Carcinoma: A Comprehensive Review

**DOI:** 10.3390/ijms27083406

**Published:** 2026-04-10

**Authors:** Dimitra Grapsa, Marina Sassi, Panagiota Mikou

**Affiliations:** Cytopathology Laboratory, “Laiko” General Hospital of Athens, 11527 Athens, Greece; dimgrap@yahoo.gr (D.G.);

**Keywords:** bladder urothelial carcinoma (BUC), diagnostic performance, fluorescence in situ hybridization (FISH), upper tract urothelial carcinoma (UTUC), urine cytology

## Abstract

Upper tract urothelial carcinoma (UTUC) is a relatively rare malignancy, far less frequent than its counterpart in the bladder, but with a more aggressive course, worse prognosis and unique diagnostic challenges. Despite the histological and molecular similarities between upper and lower tract urothelial tumours, UTUC has many key distinct traits, both clinical and genomic, and must be viewed as a separate entity from bladder urothelial carcinoma (BUC). Ureteroscopy with biopsy is the only means to obtain tissue for histo-logical confirmation of diagnosis and more accurate tumour grading, but is not always feasible or preferable because it carries the risk of potentially severe complications. Aside from the widely available but poorly sensitive urine cytology, a large variety of urine-based diagnostics are increasingly investigated as non-invasive alternatives to ureteroscopy. Fluorescence in situ hybridization (FISH) is the most widely used molecular assay for the diagnosis and monitoring of UTUC, but has failed, as of yet, to display a comparable diagnostic accuracy to the existing gold standards of computed tomography urography (CTU) and ureteroscopy. We herein aimed to comprehensively review all published data on the performance of FISH for the detection of UTUC, in comparison to urine cytology and other assays, while further commenting on the existing challenges and future perspectives in the field of urine-based diagnostics. Across all studies (*n* = 29) which were included in this review, the sensitivity and specificity of FISH ranged from 36.8% to 100.0% (mean: 75.5%; median: 78.9%) and 34.4% to 100.0% (mean: 84.9%; median: 89.9%), respectively, in the overall patient population, while in the low- versus high-grade subgroups, the sensitivity of FISH ranged from 30.0% to 90.0% (mean: 55.6%; median: 60%) versus 50.0% to 100.0% (mean: 77.9%; median: 78.8%). Furthermore, FISH showed superi-or sensitivity and similar or lower specificity in comparison to cytology, in the over-whelming majority of studies, while Xpert^®^BC Detection showed the highest sensitivity values among all evaluated assays, reaching 100% even in the low-grade subgroup, albeit at the cost of a significantly reduced specificity. Despite the adequate overall sensitivity and specificity of FISH, its suboptimal performance in the detection of low-grade UTUC seems to preclude its use as a stand-alone screening test.

## 1. Introduction

Urothelial carcinoma (UC) is a malignant neoplasm derived from the transitional epithelium (urothelium) lining the urinary tract, from the urethra and bladder (lower urinary tract) to the ureter and renal pelvis (upper urinary tract). Upper tract UC (UTUC) is a relatively rare cancer, much less frequent than its counterpart in the bladder, representing approximately 5–10% of all urothelial carcinomas and with an annual incidence rate not exceeding 2 per 100,000 person-years globally [1], with the notable exception of some endemic regions such as Eastern Asia where it comprises more than 30% of UC cases [2,3]. UTUC typically presents with hematuria, which may be visible or microscopic, and, less commonly, flank pain in the setting of urinary tract obstruction, but it may also be asymptomatic and discovered as an incidental finding on imaging or remain undiagnosed until reaching more advanced stages [4,5]. Unfortunately, 60–70% of UTUC cases fall in the latter category as they are first diagnosed at the muscle-invasive or locally advanced stage, for which treatment options are more limited and the prognosis is poor [6,7,8].

A combination of imaging studies and ureteroscopy seems to represent the theoretical gold standard with the highest sensitivity and specificity for the diagnosis of UTUC [9,10]. Computed tomography urography (CTU) is the imaging modality of choice for the diagnosis of UTUC, although it may miss carcinoma in situ (CIS) or flat tumours [9,10,11,12]. Ureteroscopy with biopsy is the only means allowing direct visualisation of the tumour and collection of tissue material for histological confirmation, subtyping and tumour grading [11,13,14], but it poses the risk of potentially severe, even life-threatening complications including ureteral perforation, infection and bleeding [11,15,16,17], while some previous reports have suggested that it may also increase the risk of intravesical recurrence (IVR) [18,19]. Furthermore, patients with UTUC are often elderly or frail and in need of frequent postoperative monitoring, due to the substantial risk of IVR and upper tract recurrence, ipsilaterally or contralaterally after kidney-saving surgery or radical nephroureterectomy, respectively [13,20,21]. Early diagnosis and close surveillance of UTUC patients, ideally using non-invasive and easily repeatable techniques, is therefore needed to reduce both the morbidity and the mortality of this aggressive malignancy.

Bladder UC (BUC) and UTUC share many common traits, including not only their common histological derivation but also some commonly shared molecular alterations driving their pathogenesis and evolution [9,22]. Interestingly, the commonly observed multifocality of UC, resulting in synchronous or metachronous involvement of multiple sites both in the upper and lower urinary tract, has been explained either as the result of intraluminal seeding of tumour cells or as a consequence of the “field effect” [23,24,25,26,27]. According to the theory of “field cancerization”, following exposure to a carcinogen, widespread genetic changes are inflicted as an early event in the urothelium of the entire urinary tract, conferring vulnerability to the development of multisite tumours and recurrences, and can be detected even in regions not showing any gross or microscopic evidence of cancer [25]. The strong histogenetic kinship between upper and lower urinary tract tumours combined with the rarity of high-quality UTUC-specific data often results in the extrapolation of findings and conclusions derived from studies on BUC to the routine care of UTUC in order to fill the existing gap [4,13,28]. This “blending” strategy is, nevertheless, far from optimal, since it seems to disregard some significant differences which also exist between these two entities. Notably, UTUC has been associated with hereditary nonpolyposis colorectal carcinoma (HNPCC), also known as Lynch syndrome, and aristolochic acid exposure, but these correlations are not revealed in BUC, suggesting that distinct molecular mechanisms and pathways are also involved in their pathogenesis and progression [22,28,29,30]. As shown by previous studies investigating the spectrum of genomic alterations in UC, although the molecular landscape in BUC and UTUC is largely similar, there are also some significant differences with regard to the frequency of several mutation patterns, such as the significantly higher prevalence of FGFR3 and HRAS mutations in UTUC versus BUC [22,31]. Furthermore, diverse mutation profiles have been observed not only among UTUC and BUC but also between ureteral and renal pelvis tumours, indicating the high molecular heterogeneity of UC [22]. According to a recent review of current international guidelines on UTUC, among all clinical guidance proposals issued by several urological and medical oncology associations and other relevant organisations, only three documents, formed by the American Urologist Association (AUA), European Association of Urology (EAU) and the National Comprehensive Cancer Network (NCCN), were found to report UTUC-specific data and address UTUC as a separate entity from BUC [4]. Often described as “disparate twins” [9,28], BC and UTUC must be viewed as similar yet essentially distinct malignancies that need to be diagnosed and managed according to disease-specific clinical data and algorithms [4,20].

Urothelial carcinomas may shed tumour cells in the urine, and thus urine-based analysis has the potential to provide diagnostically relevant tumour-specific information, offering a safe, patient-friendly and practical alternative to endoscopy. Nevertheless, intermittent rather than constant release of UC cells, often combined with the limited number of these cells amidst the remaining urine cell population, as well as the potential failure of urine tumour cell features, particularly with regard to their molecular characteristics, to accurately reflect those of the tumour tissue due to spatial tumour heterogeneity, represent some of the inherent shortcomings of urine-based testing [22,32,33]. Urine cytology is the most commonly applied urine-based diagnostic test, mainly due to its wide availability, safety, low cost and repeatability as well as its extremely high specificity and positive predictive value for the detection of high-grade UC [9,11,34]. On the other hand, its inadequate sensitivity and high interobserver variability mainly due to lack of clearly defined and reproducible criteria for the detection of low-grade UC limit its reliability as a stand-alone screening test [34,35,36]. All the above limitations are even more pronounced in the setting of UTUC, where diagnostic evaluation may be particularly challenging due to the distinct cytomorphology of high-grade tumour cells, compared to the cells derived from BUC, as well as the presence of artefacts induced by degeneration and/or instrumentation [37,38]. The sensitivity and positive predictive value (PPV) of urine cytology for low-grade disease seem to be further reduced in UTUC as compared to BUC, even when selective sampling from the upper tract instead of voided urine as well as standardised reporting such as The Paris System for Reporting Urinary Cytology (TPS) are employed [38]. Non-invasive urinary diagnostics with the potential to increase the diagnostic accuracy of UC detection, particularly for low-grade UTUC, are therefore needed to address these challenges.

Fluorescence in situ hybridization (FISH) is the most widely researched molecular assay for the diagnosis and surveillance of UTUC. The UroVysion^®^ FISH assay has been approved by the Food and Drug Administration (FDA) both for monitoring bladder cancer (BC) recurrence (since August 2001), and for the diagnostic evaluation of hematuria in patients suspected of BC (since January 2005), but not for the detection or surveillance of UTUC [39]. In addition to FISH, an increasing number of DNA- and RNA-based assays which are in various stages of development, from research use only (RUO) to the FDA-cleared and/or CE-marked status, are also being investigated for their utility in screening, diagnosis and monitoring of UTUC, following (or in parallel to) their testing in BUC [11,40,41]. Urine-based molecular tests generally display higher sensitivity compared to cytology while their results are highly subjective, reproducible and not affected by concurrent inflammatory or iatrogenic urinary tract changes, such as those induced by catheterization or intravesical Bacillus Calmette-Guerin (BCG) immunotherapy [35]. Nevertheless, none of these assays has received as of yet a regulatory clearance to be used for the diagnosis of UTUC in routine care, nor have they been included in clinical practice guidelines for UTUC, because the accumulated data not only remain limited in quantity but have also failed to support the superiority or equality of their overall performance compared to the current diagnostic gold standards of CTU and ureteroscopy, particularly in the diagnostically challenging setting of low-grade disease.

## 2. Aims and Search Strategy

We herein aimed to perform a comprehensive narrative review of the biomedical literature on the diagnostic performance of urine FISH in comparison with other assays for the detection of UTUC. For the retrieval of all relevant articles, the PubMed search engine was used, providing access to the Medline and the PubMed Central (PMC) databases up to December 2025. The search terms used were “((fluorescence in situ hybridization) AND (urine OR urinary)) AND (upper)” so as to ensure a broad coverage of the research topic. The only filter applied was for the restriction of the results to publications written in the English language only. Preprints, reviews, case reports, preclinical studies, articles with no available full text, studies with unclear inclusion criteria of patients, lack of a distinct UTUC group or not reporting any measure of the diagnostic accuracy of FISH in UTUC were excluded. The initial screening of the results was performed based on the title and abstract and, if necessary, the full text of each publication. The reference lists of all included articles were also screened for the retrieval of additional relevant studies. A total of 29 studies [42,43,44,45,46,47,48,49,50,51,52,53,54,55,56,57,58,59,60,61,62,63,64,65,66,67,68,69,70] were finally included in the present review; their results and implications are discussed below, following a brief presentation of the basic principles and historical background of FISH/UroVysion^®^ and other molecular assays which are most commonly used for the detection of UC, to provide the necessary context of information.

## 3. Overview of UroVysion^®^ and Other Commercially Available Molecular Assays

UroVysion^®^, Bladder EpiCheck^®^, Xpert^®^ Bladder Cancer Monitor (Xpert^®^ BC Monitor) and Uromonitor^®^ are, to the best of our knowledge, the only urine-based molecular assays which have received FDA approval/clearance or CE marking for their use in diagnosis and/or monitoring of UC (BC in particular) [11] and are briefly outlined below, along with other commercially available platforms.

### 3.1. FISH/UroVysion^®^

FISH is a molecular cytogenetic technique detecting the presence of chromosomal or gene abnormalities in cells with the use of fluorescently labelled DNA probes specifically designed to hybridise to the genomic target of interest (whole chromosome, chromosome arm, centromere, telomere or gene locus) through base complementary pairing [71,72]. FISH can be applied in a large variety of biological samples, including whole blood, tissue sections and cytological specimens such as the urine, and has the ability to detect multiple targets simultaneously using multiple probes labelled with different fluorophores (multicolour FISH) thus producing time- and cost-efficient results [71,73,74]. Nevertheless, FISH is a technique requiring highly specialised equipment and personnel [71] and with a steep learning curve for inexperienced observers, limiting its accessibility, while it lacks the ability, as any other urine-based diagnostic method, to accurately determine the exact tumour location [42].

Homozygous deletion of 9p21 and structural or numerical aberrations of various chromosomes, mainly including 9, 17, 7, 11 and 1, are the most frequent genetic alterations in the early stages of UC development while rarely found in normal cells [73,75,76,77]. UroVysion^®^ (Vysis-Abbott Molecular Inc., Abbott Park, IL, USA) is a multicolour FISH assay comprising a mixture of four DNA probes designed for the detection of aneuploidy of chromosomes 3, 7, 17, and loss of the 9p21 locus of the p16 gene, first validated by Sokolova et al. [73] in 2000. In the latter study, voided urine samples from patients with UC and healthy controls were tested using a FISH probe set (Vysis, Downers Grove, IL, USA), targeting the pericentromeric regions of chromosomes 3, 7, 8, 9, 11, 15, 17, 18, and Y (CEPs) and the 9p21 locus, for determination of the probe combination with the highest sensitivity for UC; the CEP7, CEP3, CEP17 and 9p21 probe subset showed the best performance in this initial cohort and was then validated in a prospective series of 179 UC patients, showing high sensitivity and specificity (84.2% and 91.8%, respectively) [73]. Following these results, the clinical use of the UroVysion^®^ assay rapidly expanded.

### 3.2. Bladder EpiCheck

Bladder EpiCheck^®^ (Nucleix, Rehovot, Israel) is a DNA platform employing reverse transcription-polymerase chain reaction (RT-PCR) for the detection of DNA methylation patterns on 15 genetic locations which are frequently altered in BC [11,78]. Briefly, following DNA extraction and separation of methylated from unmethylated DNA using a methylation-sensitive restriction enzyme, the methylated regions are amplified, detected and scored, according to the strength of the fluorescence signal emitted [11,79,80]. This test was specifically designed to be used for recurrence monitoring of patients with non-muscle invasive BC (NMIBC) [80] and has been granted FDA clearance for this exact purpose since May 2023. Other DNA methylation-based tests, including both single- or two-gene methylation markers and multi-gene panels, have also been tested and are increasingly investigated in this setting; interestingly, as previously noted in a recent meta-analysis [81], despite a seeming abundance of candidate DNA methylation tests under evaluation and some positive reports, most of these biomarkers have not been investigated in more than a single study, indicating the exploratory stage of this research field.

### 3.3. Xpert^®^ BC Monitor

Xpert^®^ BC Monitor (Cepheid, Sunnyvale, CA, USA) is a CE-marked in vitro diagnostic (IVD) employing real-time RT-PCR for the quantitative evaluation of five mRNA molecules (ABL1, CRH, IGF2, UPK1B and ANXA10) which have shown an increased expression in BC [82,83,84]. This fully automated assay was designed to be used for the early detection of recurrence in NMIBC patients after primary treatment and was first validated in a large multinational study by Wallace et al. [82] in 2018 including a training and an independent test cohort of 483 and 450 subjects, respectively; following testing of ten candidate urinary mRNA biomarkers in the training cohort, the above five-mRNA panel was identified as the best performing model and was then evaluated in the test cohort showing an overall sensitivity of 73% and specificity of 90% and 77% in the diagnostic and surveillance patient subgroups, respectively [82]. A related assay, Xpert^®^ BC Detection (Cepheid, Sunnyvale, CA, USA), using the same biomarker panel but with a different algorithm and cut-off point for positivity, specifically designed for the diagnostic setting, has also been developed for the exclusion of BC and avoidance of unnecessary cystoscopies among patients presenting with hematuria [85]. Both Xpert^®^ BC Monitor and Detection are run on the GeneXpert system, a fully automated diagnostic molecular diagnostic platform, also fully integrating all steps of the assays in a turnaround time of 90 min [84,85].

### 3.4. Uromonitor^®^

Uromonitor^®^ (U-Monitor, Porto, Portugal) is an ultra-sensitive DNA-based assay detecting UC-specific recurrent (hotspot) oncogene mutations in the TERTp and FGFR3 genes, even at levels undetectable by standard sequencing techniques [86,87,88]. More specifically, following preparation of the sample with a filtration system allowing urine cell concentration and preservation until analysis, DNA is extracted and analysed using custom-made competitive allele-specific discrimination PCR for the detection of the target mutations [86,87]. In the initial validation study of Uromonitor^®^ by Batista et al. [86], including separate cohorts for the purposes of technical and clinical validation, respectively, the assay showed adequate sensitivity (73.5%) and high specificity (93.2%) for the surveillance of NMIBC patients. In the updated version of this test (Uromonitor^®^ V.2) hotspot mutations of the KRAS gene, in addition to TERTp and FGFR3, are included in the assay to improve its diagnostic performance [87].

### 3.5. Other Commercially Available Assays

The CxBladder suite (Pacific Edge Ltd., Dunedin, New Zealand) comprises four platforms, designed for hematuria evaluation (CxBladder Triage and CxBladder Detect) [89,90], surveillance of patients with UC for potential recurrence after the initial treatment (CxBladder Monitor) [91] and the identification of UC patients with high-grade and/or late-stage disease (Cxbladder Resolve) [92]. These assays employ reverse transcription quantitative-PCR (RT-qPCR) for the quantitative evaluation of the mRNA levels of five genes, including four genes which are known to be overexpressed in UC compared to the normal urothelium (CDK1, MDK, IGFBP5 and HOXA13) as well as the CXCR2 gene which is overexpressed in neutrophils [93,94]. “Enriched” versions of these assays, designed to enhance their sensitivity, such as the Cxbladder Triage Plus, combining the standard five-gene panel included in CxBladder with six single nucleotide polymorphisms (SNPs) of the FGFR3 and TERT genes, have also been developed [95,96].

## 4. Diagnostic Performance of Urine FISH for UTUC Diagnosis

### 4.1. Methodological Aspects of Studies

The characteristics and main conclusions of all studies included in the present review are summarised in Table 1. Upon initial inspection, a significant heterogeneity between studies with regard to several methodological aspects is evident. More specifically, the study design was retrospective in 17 (17/29, 58.6%) and prospective in the remaining 12 publications (12/29, 41.4%), while a control group was included in most, but not all, retrospective cohorts (12/17. 70.6%). Furthermore, the sample size and, most importantly, the selection criteria of the patient population also differed significantly between studies, including either patients with suspected (16/29, 55.2%), confirmed UTUC (12/29, 41.4%) or both (1/29, 3.4%), while the characteristics of the control groups were also discrepant, including either healthy subjects or patients with benign urological conditions/diseases. Another major difference is the type of sample collected for analysis in each study, most commonly including voided urine (18/29, 62.1%), followed by selective upper tract washings/urine (6/29, 20.7%), mixed samples collected both from the bladder and the upper tract (4/29, 13.8%) and bladder-derived urine (1/29, 3.4%). The diagnostic methods evaluated mainly included FISH versus cytology only (20/29, 69%) or FISH versus cytology and/or other variable methods (9/29, 31%), comprising immunocytochemistry, protein-based and molecular assays. UroVysion was the assay used in the majority (15/27, 55.6%) of studies with available relevant information [42,43,44,46,47,52,53,55,59,61,62,63,64,65,67], while in the remaining studies, all originating in China, the (otherwise identical) FISH probes were supplied from other manufacturers (Beijing GP Medical Technologies, Ltd., Beijing, China or LBP Medicine Science and Technology Co., Ltd., Guangzhou, China) [45,48,49,50,51,54,56,57,60,66,68,70]. More specifically, in the majority of studies employing these alternative assays, FISH testing was conducted using commercially available multicolor, multitargeted kits (Beijing GP Medical Technologies, Ltd., Beijing, China) designed in accordance with the UroVysion probe set, often including a mix of two double-target probes, i.e., a centromere enumeration probe (CEP) for chromosomes 3 and 7 and a probe targeting the centromeric region of chromosome 17 plus locus 9p21 of the p16 gene, as previously described [50,56,70]. Notably, since all studies are listed in chronological order and although the list is far from exhaustive, including only the methods compared to FISH and not all methods which are in clinical use or under research for the detection of UC, the gradual expansion of the available urine-based assays from FISH and cytology only to more “sophisticated” molecular techniques developed in recent years is also evident.

### 4.2. FISH Versus Cytology

A total of 20 studies evaluated the performance of FISH versus cytology (17/20) [42,43,44,45,46,47,48,49,52,53,54,55,56,58,61,62,63] or with no comparator (3/20) [50,51,70] for the detection of UTUC. The diagnostic performance measures, i.e., sensitivity, specificity, positive predictive value (PPV) and negative predictive value (NPV), as reported by each study are summarised in Table 2. All four metrics were reported in 11/20 studies; in eight studies only sensitivity and specificity (7/8) or sensitivity only (1/8) were reported, while in another study specificity, PPV and NPV were reported only for high-grade tumours. Stratification of the diagnostic performance of FISH in low- and high-grade tumours was performed in four studies [44,47,58,62]. Overall, the sensitivity and specificity of FISH ranged from 51.6% to 100.0% (mean ± SD: 77.4% ± 15.7%; median: 79.5%) and 34.4% to 100.0% (mean ± SD: 84.5% ± 17.8%; median: 90.45%), respectively, while PPV and NPV ranged from 25.0% to 95.8% (mean ± SD: 77.5% ± 21.3%; median: 85.7%) and 35.0% to 100.0% (mean ± SD: 80.5% ± 21.2%; median: 87.0%), respectively. The sensitivity and specificity of cytology ranged from 18.0% to 80.0% (mean ± SD: 41.0% ± 18.3%; median: 39.0%) and 52.9% to 100.0% (mean ± SD: 89.2% ± 14%; median: 94.9%), respectively, while PPV and NPV ranged from 44.0% to 100.0% (mean ± SD: 75.6% ± 21.4%; median: 79.2%) and 49.4% to 90.3% (mean ± SD: 72.0% ± 15.4%; median: 72.2%), respectively. The sensitivity values of FISH in low- versus high-grade UTUC, evaluated in four studies, were 41.0% versus 79.0%, 60% versus 50%, 64.3% versus 86.7% and 30.0% versus 60.0%, respectively; the sensitivity values of cytology, stratified by tumour grade in three studies only, were 12.0%, 28.6% and 30.0% in the low-grade subgroup, versus 50.0%, 26.7% and 28.0% in the high-grade subgroup, respectively.

### 4.3. FISH Versus Cytology and/or Other Methods

The diagnostic performance of FISH was compared to cytology and/or variable other methods, including p16/Ki-67 dual immunostaining, the FDA-approved urine-based Nuclear Matrix Protein 22 (NMP22) test, CT imaging and molecular assays (Xpert^®^BC-Detection, Bladder EpiCheck^®^ and utLIFE) or nanotechnology-based diagnostics (UTC assay) in 9 studies [57,59,60,64,65,66,67,68,69]. The sensitivity, specificity, PPV and NPV of the above methods, as reported in each publication, are summarised in Table 3. All four metrics were reported in 5/9 studies. Overall, the sensitivity and specificity of FISH ranged from 36.8% to 92.6% (mean ± SD: 71.6% ± 19.1%; median: 78.0%) and 50.7% to 100.0% (mean ± SD: 85.6% ± 15.2%; median: 89.5%), respectively, while PPV and NPV ranged from 16.7% to 100.0% (mean ± SD: 71.0% ± 33.2%; median: 73.5%) and 56.0% to 96.2% (mean ± SD: 84.5% ± 17.1%; median: 94.1%), respectively. The sensitivity and specificity of cytology ranged from 26.3% to 74.6% (mean ± SD: 44.8% ± 18.0%; median: 42.0%) and 66.6% to 100.0% (mean ± SD: 91.7% ± 12.6%; median: 94.7%), respectively, while PPV and NPV ranged from 29.1% to 100.0% (mean ± SD: 72.0% ± 30.3%; median: 79.5%) and 50.0% to 98.0% (mean ± SD: 80.1% ± 18.8%; median: 81.4%), respectively. Xpert^®^BC-Detection was the only other assay compared to FISH in more than one study; the reported values of sensitivity and specificity for Xpert^®^BC, reported in three studies [64,65,67], were 100.0%, 90.0%, 100.0% and 16.7%, 85.0%, 4.5%, respectively; PPVs, reported in two studies [64,67], were 35.0% and 33.0% while NPVs, reported in three studies [64,65,67] were 100.0%, 98.0% and 100.0%, respectively.

In the majority (5/9) of studies, stratification of sensitivity in low- and high-grade tumours was further performed [57,64,66,67,69]. The sensitivity values of FISH in low- versus high-grade UTUC, evaluated in five studies, were 60.0% versus 69.6%, 90.0% versus 100%, 37.5% versus 78.8%, 84.6% versus 100.0% and 33.3% versus 76.9%, respectively; the sensitivity values of cytology in the low- versus high-grade subgroup, reported in four studies, were 33.3% versus 67.4%, 26.0% versus 100.0%, 30.8% versus 100.0%, and 16.7% versus 30.8%, respectively. Finally, the sensitivity of Xpert^®^BC, evaluated in low- versus high-grade UTUC in two studies, was 100.0% in both subgroups and in both studies [64,67].

### 4.4. Overall Performance of FISH

Across all studies, investigating FISH alone or with any comparator, the sensitivity and specificity of FISH in the overall patient population ranged from 36.8% to 100.0% (mean ± SD: 75.5% ± 16.8%; median: 78.9%) and 34.4% to 100.0% (mean ± SD: 84.9% ± 16.7%; median: 89.9%), respectively, while PPV and NPV ranged from 16.7% to 100.0% (mean ± SD: 75.5% ± 24.6%; median: 83.8%) and 35.0% to 100.0% (mean ± SD: 81.9% ± 19.4%; median: 81.9%), respectively. Across all studies with stratification of their results according to tumour grade, sensitivity of FISH ranged from 30.0% to 90.0% (mean ± SD: 55.6% ± 21.9%; median: 60.0%) in the low-grade subgroup and from 50.0% to 100.0% (mean ± SD: 77.9% ± 16.7%; median: 78.8%) in the high-grade subgroup.

Although not directly comparable to pooled estimates, the sensitivity and specificity values of FISH for the detection of UTUC, as herein evaluated, are well within the range of the results of two previous meta-analyses [97,98]. More specifically, Jin et al. [97] reported pooled sensitivities and specificities of 84.0% and 89.5% for FISH versus 40% and 95.9% for cytology, respectively, while the respective sensitivity and specificity values reported for FISH by Aalami AH et al. [98] were 72% and 95%, respectively.

## 5. Discussion

Despite the significant methodological differences observed between studies evaluating the diagnostic performance of FISH in UTUC, and the wide range of sensitivity and specificity values reported, as analysed above, their conclusions, for the most part, are surprisingly consistent. FISH showed superior sensitivity and NPV and equal or lower specificity compared to cytology for the detection of UTUC in all studies evaluating these two methods, with the exception of the report by Freund et al. [61]; in the latter study, the specificity of cytology was slightly lower compared to FISH (80% vs. 90%, respectively), but this might be attributed to the modified FISH protocol employed (the FISH assay was performed in 1 mL of urine instead of the minimum urine volume of 32 mL advised by the manufacturer) as well as the small sample size of the patient population enrolled (10 cases only) [61]. The combination of FISH and cytology, evaluated in a retrospective [49] and two prospective UTUC cohorts [52,53], showed even higher sensitivity compared to FISH alone in two of these studies (85.9% versus 78.9% and 100.0% versus 84.2%, respectively) [49,53] while in the third study cyto-FISH had lower sensitivity to FISH when hypertetrasomy only (defined as more than four signals per probe per nuclei) was considered abnormal and higher when both tetrasomy (HT) and hyper-HT FISH results were considered positive; in the latter case, a parallel decrease in specificity from 84% to 53% was nevertheless observed [52]. Based on these findings, the authors advised against interpreting tetrasomy FISH results as abnormal, to avoid reduction in the assay specificity, while also emphasising the importance of standardised interpretation criteria for the FISH assay [52].

Another consistent observation among studies was the significantly higher sensitivity of FISH (and cytology) for the detection of high- versus low-grade UTUC. Except for one study, by Johannes et al. [47], showing a slightly increased sensitivity of FISH for low- versus high-grade UTUC (60% vs. 50%), all other studies including a stratified analysis according to tumour grade confirmed the superior performance of FISH for the detection of high-grade disease. The inability of FISH to detect a significant percentage of low-grade tumours may be most likely due, as previously hypothesised, to their frequent near-normal ploidy, resembling normal epithelial cells [97,99,100] or to the reduced propensity of low-grade tumours to shed cells in the urine [66,101]. Using urine derived directly from the upper tract, i.e., via ureteral catheterization and other techniques, may increase the sensitivity of FISH for the detection of low-grade UTUC, as shown in the studies by Pycha et al. [67] and D’Elia et al. [64], although the need for invasive sampling and its associated risks and discomfort for the patient negate the advantage of urine-based testing as a non-invasive diagnostic modality. Bier et al. [59] reported an increased sensitivity of FISH (and cytology) when urine was derived from the upper tract instead of the bladder, albeit at the cost of a reduced specificity, in contrast to previous findings by Mian et al. [46] and Akkad et al. [43] showing higher specificity when upper tract samples were used; this was explained by the authors as the potential result of upper tract manipulation, leading to mechanically induced chromosomal aberrations and/or the different cut-offs for FISH positivity employed in their study [59].

Although sensitivity and specificity are, by definition, prevalence-independent, reflecting the most representative measures of the intrinsic accuracy of any index diagnostic test, clinical experience has shown that these metrics may also be affected by the characteristics of the evaluable population [102]. In settings (or study cohorts) with low prevalence of disease, i.e., when FISH is used for triage of patients with hematuria, specificity may be higher, approximating 100%, while in cohorts composed of patients with a previous diagnosis of UTUC the specificity values may be considerably lower, given the low probability of true negative results, as previously emphasised by Chen and Grasso [44]. Huang et al. [50] reported 100% sensitivity and PPV and high specificity (99.3%) for FISH when it was evaluated as a screening tool for the detection of UTUC among a large prospective cohort of patients (*n* = 285) with asymptomatic hematuria and negative urine cytology, suggesting that the performance of FISH may be optimal when used in carefully selected populations. Furthermore, in cohorts with a lower proportion of high-grade tumours the overall sensitivity of FISH may be lower compared to those with a higher frequency of high-grade disease, suggesting the need for stratifying the diagnostic accuracy measures by tumour grade [54,55,58]. In a retrospective study by Gomella et al. [55], investigating the performance of FISH versus cytology among patients with suspected UC, the sensitivity of FISH was significantly lower as compared to an earlier study by Xu et al. [49] (54.9% versus 78.9%); this discrepancy was explained by the authors as a potential consequence of the distinct composition of their patient population, mainly comprising patients managed endoscopically due to low-grade and low-volume disease [55], sharply contrasting the aggressively treated high-grade tumours which were mainly included in the other cohort [49].

Given the suboptimal specificity of FISH as well as the probability of sample “contamination” from concurrent BUC, especially in patients with a previous history of BC, positive FISH results must be confirmed with biopsy and imaging prior to therapeutic intervention [52,55]. On the other hand, some of the apparently “false-positive” FISH results, i.e., positive FISH in patients with no imaging or histological evidence of malignancy, may not necessarily reflect an inadequate specificity, but may instead represent the so-called “anticipatory positives”, where, presumably, the chromosomal alterations seem to precede—and predict—the clinical, radiological and/or macroscopic appearance of UC by several months [61,103,104]. Although this phenomenon has been described as of yet only in BUC, it may also be applicable to UTUC as well, as previously hypothesised [55]. Furthermore, although it remains largely unknown whether these anticipatory positives represent tumours that are truly undetectable or simply missed on previous imaging and/or endoscopy [105], either way, this fact dictates the need for close monitoring of patients with positive FISH results even when the remaining diagnostic tests are negative.

In the present review, comparison of FISH with other molecular assays, including Xpert^®^BC-Detection, Bladder Epicheck^®^, a DNA methylation panel, an NGS platform (utLIFE) and a nanotechnology-based assay (UTC assay), showed, in all studies, an increased sensitivity of these methods compared to FISH, which was nevertheless also accompanied, in almost all cases, by a significant reduction in specificity. More specifically, the highest sensitivity values, reaching 100%, both in the overall patient population and in the low- and high-grade subgroups, were reported for Xpert^®^BC Detection in two prospective studies of patients with clinical suspicion of UTUC, performed by D’Elia et al. [64] and Pycha et al. [67], along with plummeting of overall specificity values to 16.7% and 4.5%, respectively. In contrast to these findings, a larger prospective study by Kavcic et al. [65] including a mixed population of patients monitored or suspected for UTUC, showed far more balanced sensitivity and specificity values for the same assay (90% and 85%, respectively). Furthermore, the UTC assay, although succeeding in outperforming the sensitivity of FISH (85.0% versus 73.0%) while maintaining a particularly high specificity of 92.5%, failed to reach equally high levels of sensitivity compared to the Xpert^®^BC platform [66]. Admittedly, since the primary aim of this review was to investigate the diagnostic performance of the FISH assay, thus including other molecular assays only when these were compared to FISH, the above results are merely a glimpse of the entire spectrum of all urine-based molecular diagnostics available and cannot be used as a representative indicator of their diagnostic accuracy for the detection and monitoring of UTUC.

The diagnostic value of any candidate biomarker must always be evaluated within the intended clinical context of use. Biomarkers with high sensitivity and NPV, such as the highly sensitive Xpert^®^BC platform, might be suitable for ruling out the possibility of UC and reduce the frequency of unnecessary invasive procedures, as shown in a recent study [64], but not to confirm the presence of disease, given the increased possibility of a false-positive result. In contrast, biomarkers with higher specificity and PPV, such as cytology, may be more useful as confirmatory tests among patients with high clinical and/or imaging suspicion of UTUC. Based on the current data, although FISH shows an excellent sensitivity and specificity for the detection of high-grade UTUC, its low accuracy in detecting low-grade tumours seems to preclude its use as a stand-alone screening test.

## 6. Limitations

Before drawing the major conclusions, some limitations of the present work must be briefly discussed. First, this was a narrative and not a systematic review; therefore, although selected characteristics of each study, such as the inclusion criteria of patients, the study design, the sampling methods and, most importantly, the exact values of sensitivity, specificity, PPV and NPV were extracted, listed and compared between studies, this was not done according to a predefined protocol and was not followed by data synthesis and meta-analysis. Furthermore, FISH was the only molecular method that was comprehensively reviewed and discussed herein, and the interested reader is referred to other reviews for a more detailed overview of all molecular assays which are commercially available or in development for the detection of UC [9,11,32,40,41]. Finally, the only databases used for the retrieval of all relevant publications were MEDLINE and PubMed Central, as the most representative and inclusive of high-quality biomedical evidence, and therefore it is possible that some additional results indexed in other databases may have been missed.

## 7. Conclusions and Perspectives

UTUC is not only the disparate but also the orphan twin of BUC. Too rare to permit gathering of adequate and robust real-world data but also deadly enough to lead to a significant mortality, this largely understudied malignancy remains challenging both to diagnose early and treat effectively. Given its deep anatomic location, requiring the performance of ureteroscopy with biopsy for tissue collection, histological classification and tumour grading, a variety of non-invasive urine-based diagnostics, including molecular biomarkers, are increasingly investigated for its detection and monitoring. Urine FISH remains the most widely used molecular technique in the setting of UTUC, most commonly in combination with cytology. Despite the high sensitivity and specificity of FISH for the detection of high-grade UTUC, its inability to detect a significant percentage of low-grade disease as well as to determine the tumour location hinder its use as a sole screening test. Newer molecular assays, such as the Xpert^®^BC platform, seem to increase the sensitivity of detection for low-grade disease, albeit with the collateral damage of a significantly decreased specificity. Most importantly, none of these non-invasive alternatives have shown, as of yet, an overall diagnostic accuracy comparable to the gold standards of ureteroscopy and CTU, and their use remains confined mostly to adjunctive testing.

Although large comparative prospective studies complemented with external validation are indispensable to more accurately evaluate the diagnostic performance of FISH and other molecular assays for the detection of UTUC, the rarity of this disease may render this goal almost unattainable. Systematic reviews with network meta-analyses, for simultaneous evaluation of multiple biomarkers, might substitute for the paucity of robust original research studies and are eagerly awaited. Furthermore, focusing our research interest towards investigating and validating panels of multiple biomarkers designed to meet specific clinical needs (i.e., screening of asymptomatic patients vs. triage of patients with hematuria vs. monitoring of patients with confirmed disease) might be a more fruitful approach than trying to find the one-size-fits-all assay to cover all settings. A multi-omics approach, in this regard, combining protein-, genetic- and epigenetic-based biomarkers, may be more effective, as previously proposed [9,106,107], although the cost-effectiveness of these complex diagnostic models must be carefully assessed as well.

As insightfully commented by Satyal et al. [108] in a review of recent advancements in urine-based liquid biopsies, “The market is littered with high performing tests that never gained traction because they did not address a specific clinical need (or at least do not address it unambiguously), were too expensive, or only add an incremental amount of information to the clinical decision-making process”. The impressive body of knowledge and continuously evolving possibilities now offered to us by the recent advances in massively parallel DNA sequencing technologies, further empowered by their increasing availability and reduced costs, as well as by the expanding clinically oriented applications of nanotechnology and artificial intelligence, must be carefully matched to real-world clinical needs and expectations. Most importantly, one may argue that our struggle to replace the current diagnostic gold standards with a non-invasive surrogate may prove to be not only unrealistic but also counter-productive. Similarly to a previous proposal for BUC [109], integration of the most accurate and clinically relevant molecular biomarkers into UTUC-specific and risk-stratified algorithms, combined with the “traditional” diagnostic tools of imaging, cytology and endoscopy, as indicated in each clinical scenario, may represent the best balancing act between the desired enhancement of diagnostic efficacy and the unwanted increase in procedure-related morbidity and costs. Hopefully, the accelerating progress in the research field of biomarker-driven diagnostics might provide in the near future a more solid ground to stand upon and envision the best path forward.

## Figures and Tables

**Table 1:** Characteristics and main conclusions of studies evaluating the performance of urine FISH versus other methods for the diagnosis of upper tract urothelial carcinoma.

Authors (Year) *	Patient Population (*n*) **	Study Design	Specimen Type	Diagnostic Methods Evaluated	Conclusions
Marín-Aguilera et al. (2007) [42]	Pts with confirmed UTUC (*n* = 30)	▪Retrospective (Case–control) ▪Prospective enrollment	Voided urine	▪FISH (UroVysion) ▪Cytology	FISH showed superior sensitivity and similar specificity compared to cytology.
Akkad et al. (2007) [43]	Pts with suspected UTUC (*n* = 16)	Prospective	▪Voided urine ▪Upper tract washings	▪FISH (UroVysion) ▪Cytology	FISH showed superior sensitivity and equal specificity compared to cytology.
Chen et al. (2008) [44]	Pts monitored for UTUC (*n* = 43)	Retrospective	▪Voided urine ▪Bladder washings ▪Upper tract washings	▪FISH (UroVysion) ▪Cytology	FISH showed superior overall sensitivity compared to cytology. Both FISH and cytology had higher sensitivity for high- vs. low-grade tumours.
Luo et al. (2009) [45]	Pts with confirmed UTUC (*n* = 21)	Retrospective (Case–control)	Voided urine	▪FISH ▪Cytology	FISH showed superior sensitivity and equal specificity compared to cytology.
Mian et al. (2010) [46]	Pts with suspected UTUC (*n* = 55)	Prospective	Upper tract washings	▪FISH (UroVysion) ▪Cytology	FISH showed superior sensitivity and similar specificity compared to cytology.
Johannes et al. (2010) [47]	Pts monitored for UTUC (*n* = 35)	Retrospective	Voided urine	▪FISH (UroVysion) ▪Cytology	FISH showed superior sensitivity and lower specificity compared to cytology.
Shan et al. (2010) [48]	Pts with confirmed UTUC (*n* = 50)	Retrospective (Case–control)	Voided urine	▪FISH ▪Cytology	FISH showed superior sensitivity and equal specificity compared to cytology.
Xu et al. (2011) [49]	Pts with confirmed UTUC (*n* = 71)	Retrospective (Case–control)	Voided urine	▪FISH ▪Cytology ▪Cyto-FISH (Cytology + FISH)	Cyto-FISH showed significantly superior sensitivity compared to cytology and slightly superior sensitivity compared to FISH. Specificities of all methods were similar.
Huang et al. (2012) [50]	Pts with asymptomatic hematuria and negative urine cytology (*n* = 285)	Prospective	Voided urine	FISH	FISH showed extremely high sensitivity and specificity among pts with clinical suspicion of UTUC and negative cytology.
Wang et al. (2012) [51]	Pts with hematuria due to UTUC (*n* = 34)	Retrospective (Case–control)	Voided urine	FISH	FISH may be useful for distinguishing benign hematuria from hematuria due to UTUC.
Reynolds et al. (2014) [52]	Pts with suspected UTUC (*n* = 61)	Prospective	Upper tract washings	▪FISH HT (UroVysion) ▪FISH tetrasomy + HT ▪Cytology ▪Cytology + FISH HT	FISH showed superior sensitivity and lower specificity compared to cytology. Interpreting tetrasomy FISH results as abnormal lowered the assay specificity.
Gruschwitz et al. (2014) [53]	Pts with suspected UTUC (*n* = 82)	Prospective	Upper tract washings	▪FISH (UroVysion) ▪Cytology ▪Cytology + FISH	FISH showed superior sensitivity and equal specificity compared to cytology. FISH and cytology combination has the highest sensitivity.
Yu et al. (2016) [54]	Pts with suspected UTUC (*n* = 125)	▪Retrospective (Case–control) ▪Prospective enrollment	Voided urine	▪FISH ▪Cytology	FISH showed superior sensitivity and lower specificity and PPV compared to cytology.
Gomella et al. (2017) [55]	Pts with suspected UTUC or BC (*n* = 415)	Retrospective	Voided urine	▪FISH (UroVysion) ▪Cytology	FISH showed slightly higher sensitivity and similar specificity to cytology.
Lin et al. (2017) [56]	Pts with suspected UTUC or BC (*n* = 119)	Prospective	Voided urine	▪FISH ▪Cytology	FISH showed significantly superior sensitivity compared to cytology.
Sun et al. (2017) [57]	Pts with confirmed UTUC (*n* = 61)	Retrospective (Case–control)	Voided urine	▪FISH ▪Cytology ▪p16/Ki-67 staining	FISH showed superior sensitivity compared to cytology and p16/Ki-67 staining for the detection of LG-UTUC.
Yu et al. (2017) [58]	Pts with suspected UTUC (*n* = 52)	Prospective	Voided urine	▪FISH ▪Cytology	FISH showed superior sensitivity and similar specificity compared to cytology.
Bier et al. (2018) [59]	Pts with suspected UTUC (*n* = 682)	Retrospective	▪Upper tract-derived urine ▪Bladder-derived urine	▪FISH (UroVysion) ▪Cytology ▪Immunocytology ▪NMP22 ELISA	The sensitivity of urine biomarkers for the detection of UTUC may be higher in urine collected from the upper urinary tract compared to the bladder.
Yang et al. (2018) [60]	Pts with suspected UTUC (*n* = 30)	Prospective	Voided urine	▪FISH ▪Cytology ▪CT scan	FISH showed superior sensitivity and lower specificity compared to cytology.
Freund et al. (2019) [61]	Pts with confirmed UTUC (*n* = 10)	Retrospective (Case-control)	Urine sampling with ureteral splint	▪FISH in 1 mL of urine (UroVysion) ▪Cytology	FISH in 1 mL of selective upper tract urine showed adequate sensitivity and specificity.
Sassa et al. (2019) [62]	Pts with suspected UTUC (*n* = 112)	▪Retrospective (Case–control) ▪Prospective enrollment	▪Ureteral-selected urine ▪Voided urine	▪FISH (UroVysion) ▪Cytology	FISH showed superior sensitivity and lower specificity compared to cytology for the detection of HG-UTUC. Both methods showed low sensitivity for the detection of LG-UTUC.
Iwata et al. (2021) [63]	Pts with history of RNU for UTUC, monitored for IVR (*n* = 65)	Prospective	Bladder-derived urine (immediately after RNU and 5 days later)	▪FISH (UroVysion) ▪Cytology	FISH performed in multiple urine samples may show increased sensitivity compared to cytology for the detection of IVR after RNU.
D’Elia et al. (2022) [64]	Pts with suspected UTUC (*n* = 82)	Prospective	Upper tract-derived urine	▪FISH (UroVysion) ▪Cytology ▪Xpert^®^BC-Detection	FISH and Xpert^®^BC-Detection showed superior sensitivity compared to cytology.
Kavcic et al. (2022) [65]	Pts with suspected UTUC or monitored for UTUC (*n* = 204)	Prospective	Voided urine	▪FISH (UroVysion) ▪Xpert^®^BC-Detection	FISH showed lower sensitivity and higher specificity compared to Xpert^®^BC-Detection.
Wang et al. (2022) [66]	Pts with suspected UTUC (*n* = 90)	Retrospective (Case–control)	Voided urine	▪FISH ▪UTC assay	UTC assay showed higher sensitivity compared to FISH for the detection of UTUC, particularly LG-UTUC.
Pycha et al. (2023) [67]	Pts with suspected UTUC (*n* = 79)	Prospective	Ureteral-selected urine	▪FISH (UroVysion) ▪Cytology ▪Xpert^®^BC-Detection ▪Bladder Epicheck	FISH showed lower sensitivity compared to Xpert^®^BC-Detection but higher than Bladder Epicheck. Cytology showed the highest specificity of all, followed by FISH.
Wei et al. (2024) [68]	Pts with confirmed UTUC (*n* = 37)	Retrospective (Case–control)	Voided urine	▪FISH ▪Cytology ▪Cytology + FISH ▪DNA methylation testing	DNA methylation testing showed the highest sensitivity and NPV compared to FISH and other methods. Specificities of all methods were equal.
Zuo et al. (2024) [69]	Pts monitored for UTUC (*n* = 93)	▪Retrospective (Case–control) ▪Prospective enrollment	Voided urine	▪FISH ▪Cytology ▪utLIFE	utLIFE showed superior sensitivity compared to FISH and cytology, particularly among pts with LG-UTUC.
Xu et al. (2025) [70]	Pts with suspected UC (*n* = 215)	Retrospective	Voided urine	FISH	FISH showed similar sensitivity and specificity for the detection of BC vs. UTUC.

* Studies are listed in chronological order of publication; ** initially enrolled patients; BC: bladder cancer; CT: computed tomography; FISH: fluorescence in situ hybridization; HG: high-grade; HT: hypertetrasomy; IVR: intravesical recurrence; LG: low-grade; NMP22: nuclear matrix protein 22; pts: patients; RNU: radical nephroureterectomy; UTUC: upper tract urothelial carcinoma.

**Table 2:** Diagnostic performance of urine FISH versus cytology for the detection of upper tract urothelial carcinoma.

Authors (Year) *	Diagnostic Methods Evaluated	Sensitivity (%)	Specificity (%)	PPV (%)	NPV (%)
Marín-Aguilera et al. (2007) [42]	▪FISH	76.7	94.7	95.8	72.0
	▪Cytology	36.0	100.0	100.0	54.0
Akkad et al. (2007) [43]	▪FISH	87.5	80.0	87.5	80.0
	▪Cytology	60.0	80.0	75.0	66.0
Chen et al. (2008) [44]	Overall				
	▪FISH	52.0	63.0	N/A	N/A
	▪Cytology	24.0	79.0	N/A	N/A
	Low-grade tumours				
	▪FISH	41.0	N/A	N/A	N/A
	▪Cytology	12.0	N/A	N/A	N/A
	High-grade tumours				
	▪FISH	79.0	N/A	N/A	N/A
	▪Cytology	50.0	N/A	N/A	N/A
Luo et al. (2009) [45]	▪FISH	85.7	100.0	N/A	N/A
	▪Cytology	23.8	100.0	N/A	N/A
Mian et al. (2010) [46]	▪FISH	100.0	89.5	85.7	100.0
	▪Cytology	20.8	97.4	83.3	66.1
Johannes et al. (2010) [47]	Overall				
	▪FISH	54.0	78.0	88.0	35.0
	▪Cytology	18.0	100.0	Ν/A	Ν/A
	Low-grade tumours				
	▪FISH	60.0	N/A	N/A	N/A
	▪Cytology	N/A	N/A	N/A	N/A
	High-grade tumours				
	▪FISH	50.0	N/A	N/A	N/A
	▪Cytology	N/A	N/A	N/A	N/A
Shan et al. (2010) [48]	▪FISH	84.0	96.0	N/A	N/A
	▪Cytology	40.0	96.0	N/A	N/A
Xu et al. (2011) [49]	▪FISH	78.9	97.8	N/A	N/A
	▪Cytology	45.1	100.0	N/A	N/A
	▪Cyto-FISH (Cytology + FISH)	85.9	97.8	N/A	N/A
Huang et al. (2012) [50]	FISH	100.0	99.3	81.8	100.0
Wang et al. (2012) [51]	FISH	73.5	93.9	92.6	77.5
Reynolds et al. (2014) [52]	Overall				
	▪FISH hypertetrasomy (HT)	43	84	38	86
	▪FISH tetrasomy and HT	67	53	25	87
	▪Cytology	38	89	44	86
	▪Cytology + FISH HT	52	77	34	88
	After exclusion of cases with concurrent or prior BC				
	▪FISH hypertetrasomy (HT)	50	89	54	88
	▪FISH tetrasomy and HT	79	52	29	91
	▪Cytology	50	95	70	88
	▪Cytology + FISH HT	64	84	50	90
Gruschwitz et al. (2014) [53]	▪FISH	84.2	91.1	76.2	94.4
	▪Cytology	52.6	91.4	66.7	85.5
	▪Cytology + FISH	100.0	83.6	N/A	N/A
Yu et al. (2016) [54]	▪FISH	84.2	89.8	80.0	97.8
	▪Cytology	42.1	94.9	100.0	90.3
Gomella et al. (2017) [55]	▪FISH	51.9	89.3	90.0	50.0
	▪Cytology	42.2	90.2	87.3	49.4
Lin et al. (2017) [56]	▪FISH	73.7	N/A	N/A	N/A
	▪Cytology	31.6	N/A	N/A	N/A
Yu et al. (2017) [58]	**Overall**				
	▪FISH	79.5	96.2	N/A	N/A
	▪Cytology	27.3	100.0	N/A	N/A
	Low-grade tumours				
	▪FISH	64.3	N/A	N/A	N/A
	▪Cytology	28.6	N/A	N/A	N/A
	High-grade tumours				
	▪FISH	86.7	N/A	N/A	N/A
	▪Cytology	26.7	N/A	N/A	N/A
Freund et al. (2019) [61]	▪FISH in 1 mL of urine	90.0	80.0	N/A	N/A
	▪Cytology	80.0	67.0	N/A	N/A
Sassa et al. (2019) [62]	Low-grade tumours				
	▪FISH	30.0	N/A	N/A	N/A
	▪Cytology	30.0	N/A	N/A	N/A
	High-grade tumours				
	▪FISH	60.0	84.0	93.8	41.2
	▪Cytology	28.0	100.0	100.0	31.6
Iwata et al. (2021) [63]	▪FISH	95.5	34.4	50.0	91.7
	▪Cytology	75.0	52.9	48.4	78.3
Xu et al. (2025) [70]	FISH	51.6	95.8	N/A	N/A

* Studies are listed in chronological order of publication; BC: bladder cancer; FISH: fluorescence in situ hybridization; NPV: negative predictive value; PPV: positive predictive value.

**Table 3:** Diagnostic performance of urine FISH versus cytology and/or other methods for the detection of upper tract urothelial carcinoma.

Authors (Year) *	Diagnostic Methods Evaluated	Sensitivity (%)	Specificity (%)	PPV (%)	NPV (%)
Sun et al. (2017) [57]	Overall				
	▪FISH	67.2	90.0	N/A	N/A
	▪Cytology	59.0	94.4	N/A	N/A
	▪p16/Ki-67 dual immunostaining	53.1	100.0	N/A	N/A
	Low-grade tumours				
	▪FISH	60.0	N/A	N/A	N/A
	▪Cytology	33.3	N/A	N/A	N/A
	▪p16/Ki-67 dual immunostaining	12.5	N/A	N/A	N/A
	High-grade tumours				
	▪FISH	69.6	N/A	N/A	N/A
	▪Cytology	67.4	N/A	N/A	N/A
	▪p16/Ki-67 dual immunostaining	66.7	N/A	N/A	N/A
Bier et al. (2018) [59]	▪Bladder-derived urine				
	▪FISH	52.9	85.0	19.6	96.3
	▪Cytology	59.3	82.9	22.2	96.1
	▪Immunocytology	50.0	69.8	10.5	95.2
	▪NMP22 ELISA	62.5	31.3	6.1	92.1
	Upper tract-derived urine				
	▪FISH	79.0	50.7	16.7	95.1
	▪Cytology	74.6	66.6	29.1	93.4
	▪Immunocytology	100.0	66.7	25.0	100.0
	▪NMP22 ELISA	100.0	5.9	15.8	100.0
Yang et al. (2018) [60]	▪FISH	85.7	88.9	N/A	N/A
	▪Cytology	28.6	100.0	N/A	N/A
	▪CT scan	66.7	77.8	N/A	N/A
D’Elia et al. (2022) [64]	Overall				
	▪FISH (UroVysion)	92.6	85.0	73.5	96.2
	▪Cytology	51.9	95.0	82.4	81.4
	▪Xpert^®^BC-Detection	100.0	16.7	35.0	100.0
	Low-grade tumours				
	▪FISH (UroVysion)	90.0	N/A	N/A	N/A
	▪Cytology	26.0	N/A	N/A	N/A
	▪Xpert^®^BC-Detection	100.0	N/A	N/A	N/A
	High-grade tumours				
	▪FISH (UroVysion)	100.0	N/A	N/A	N/A
	▪Cytology	100.0	N/A	N/A	N/A
	▪Xpert^®^BC-Detection	100.0	N/A	N/A	N/A
Kavcic et al. (2022) [65]	▪FISH (UroVysion)	78.0	93.0	N/A	96.0
	▪Xpert^®^BC-Detection	90.0	85.0	N/A	98.0
Wang et al. (2022) [66]	Overall				
	▪FISH (UroVysion)	73.3	95.0	95.7	70.4
	▪UTC assay	85.0	92.5	94.4	80.4
	Low-grade tumours				
	▪FISH (UroVysion)	37.5	N/A	N/A	N/A
	▪UTC assay	87.5	N/A	N/A	N/A
	High-grade tumours				
	▪FISH (UroVysion)	78.8	N/A	N/A	N/A
	▪UTC assay	84.6	N/A	N/A	N/A
Pycha et al. (2023) [67]	Overall				
	▪FISH	87.1	81.8	69.2	93.1
	▪Cytology	41.9	93.9	76.5	77.5
	▪Xpert^®^BC-Detection	100.0	4.5	33.3	100.0
	▪Bladder Epicheck	64.5	78.8	58.8	82.5
	Low-grade tumours				
	▪FISH	84.6	N/A	N/A	N/A
	▪Cytology	30.8	N/A	N/A	N/A
	▪Xpert^®^BC-Detection	100.0	N/A	N/A	N/A
	▪Bladder Epicheck	57.7	N/A	N/A	N/A
	High-grade tumours				
	▪FISH	100.0	N/A	N/A	N/A
	▪Cytology	100.0	N/A	N/A	N/A
	▪Xpert^®^BC-Detection	100.0	N/A	N/A	N/A
	▪Bladder Epicheck	100.0	N/A	N/A	N/A
Wei et al. (2024) [68]	▪FISH	45.0	100.0	100.0	56.0
	▪Cytology	31.0	100.0	100.0	50.0
	▪Cytology +FISH	66.0	100.0	100.0	67.0
	▪DNA methylation testing	76.0	100.0	100.0	74.0
Zuo et al. (2024) [69]	Overall				
	▪FISH	36.8	N/A	N/A	N/A
	▪Cytology	26.3	N/A	N/A	N/A
	▪utLIFE	94.7/96.8 **	96.3 **	96.8 **	96.3 **
	Low-grade tumours				
	▪FISH	33.3	N/A	N/A	N/A
	▪Cytology	16.7	N/A	N/A	N/A
	▪utLIFE	100.0	N/A	N/A	N/A
	High-grade tumours				
	▪FISH	76.9	N/A	N/A	N/A
	▪Cytology	30.8	N/A	N/A	N/A
	▪utLIFE	92.3	N/A	N/A	N/A

* Studies are listed in chronological order of publication; ** Different data set; FISH: fluorescence in situ hybridization; NPV: negative predictive value; PPV: positive predictive value.

## Data Availability

No new data were created or analyzed in this study. Data sharing is not applicable to this article.
